# Comparative risk of systemic autoimmune diseases in juvenile idiopathic arthritis treated with TNF-α or IL-6 inhibitors: a real-world cohort study

**DOI:** 10.3389/fimmu.2025.1744226

**Published:** 2026-01-13

**Authors:** Jih-Jin Tsai, Li-Teh Liu, Ping-Chang Lin, Yu-Hsun Wang, Yung-Heng Lee, James Cheng-Chung Wei

**Affiliations:** 1 Tropical Medicine Center, Kaohsiung Medical University Hospital, Kaohsiung, Taiwan; 2 Division of Infectious Diseases, Department of Internal Medicine, Kaohsiung Medical University Hospital, Kaohsiung, Taiwan; 3 School of Medicine, College of Medicine, Kaohsiung Medical University, Kaohsiung, Taiwan; 4 Department of Medical Laboratory Science and Biotechnology, College of Medical Technology, Chung Hwa University of Medical Technology, Tainan, Taiwan; 5 Department of Medical Research, Chung Shan Medical University Hospital, Taichung, Taiwan; 6 Institute of Medicine, Chung Shan Medical University, Taichung, Taiwan; 7 Department of Orthopedics, Feng Yuan Hospital, Ministry of Health and Welfare, Taichung, Taiwan; 8 Institute of Medical Science and Technology, National Sun Yat-sen University, Kaohsiung, Taiwan; 9 Department of Senior Services Industry Management, Minghsin University of Science and Technology, Hsinchu, Taiwan; 10 Department of Recreation and Sport Management, Shu-Te University, Kaohsiung, Taiwan; 11 Department of Allergy, Immunology & Rheumatology, Chung Shan Medical University Hospital, Taichung, Taiwan; 12 Office of Research and Development, Asia University, Taichung, Taiwan; 13 Graduate Institute of Integrated Medicine, China Medical University, Taichung, Taiwan

**Keywords:** interleukin-6 inhibitors, juvenile idiopathic arthritis, pediatric, systemic autoimmune diseases, tumor necrosis factor inhibitors

## Abstract

**Objective:**

This study aimed to compare the risk of developing systemic autoimmune diseases (SADs) among pediatric patients with juvenile idiopathic arthritis (JIA) treated with tumor necrosis factor inhibitors (TNFi) versus interleukin-6 inhibitors (IL-6i), based on real-world data.

**Methods:**

We conducted a retrospective real-world cohort study using the TriNetX Research Network, which contains data from over 122 million patients. Individuals ≤18 years of age with a diagnosis of JIA who initiated TNFi or IL-6i therapy between January 1, 2013, and December 31, 2024, were included. Propensity score matching (1:1) was performed to balance baseline characteristics. The primary outcome was the incidence of SADs. Hazard ratios (HRs) were estimated using Cox proportional hazards models, and subgroup and sensitivity analyses were conducted to assess robustness.

**Results:**

After matching, 1,192 patients were included in each cohort. The TNFi group demonstrated a significantly lower incidence of SADs than the IL-6i group (17 vs. 45 events; HR = 0.37, 95% CI: 0.20–0.63). Subgroup analyses showed consistent protective effects of TNFi across age, sex, and concomitant medication strata. Sensitivity analyses across three adjusted models confirmed the robustness of the findings, yielding HRs ranging from 0.28 to 0.46.

**Conclusion:**

Among pediatric patients with JIA, TNF inhibitor therapy was associated with a substantially lower risk of developing systemic autoimmune diseases compared with IL-6 inhibitor therapy. These findings may inform biologic selection and long-term safety considerations in pediatric rheumatologic practice.

## 1 Introduction

Juvenile idiopathic arthritis (JIA) is a chronic, heterogeneous autoimmune disease and also the most common rheumatologic disease of childhood (1) that affects approximately 1 in 1,000 children (1, 2). JIA is characterized by pain, stiffness, joint damage, growth abnormalities, and poses substantial risks to physical function and quality of life (3, 4). The classification criteria include the revised International League of Associations for Rheumatology (ILAR) criteria (5) and the Pediatric Rheumatology International Trial Organization (PRINTO) (6, 7). Both criteria differ in different categories and different ages of onset of symptoms before the 16th year of age (ILAR) and 18th year of age (PRINTO). Among the various therapeutic strategies, biologic disease-modifying antirheumatic drugs (bDMARDs) such as tumor necrosis factor inhibitors (TNFi) and interleukin-6 inhibitors (IL6i) have revolutionized the management of JIA, especially in patients refractory to conventional treatments (8–10).

Despite their clinical efficacy, these immunomodulators have raised concerns about paradoxical autoimmune effects (11–14), particularly the development of systemic autoimmune diseases (SADs) such as systemic lupus erythematosus (SLE) (15), autoimmune renal disorders (16), and vasculitis (17). While these risks have been studied in adult populations, comparative safety profiles of TNFi and IL6i in pediatric JA patients remain poorly understood, creating a critical gap in evidence-based pediatric rheumatologic care.

Juvenile idiopathic arthritis (JIA) includes several subtypes that differ in clinical manifestations, treatment responses, and long-term outcomes. TNFi agents—including etanercept, adalimumab, and infliximab—are frequently used as first-line biologics to control inflammation by neutralizing TNF-α, a cytokine central to the pathogenesis of synovitis and joint destruction (8, 10, 18) IL6i therapies, such as tocilizumab, offer an alternative, particularly in systemic JIA, by inhibiting IL-6-mediated pathways implicated in systemic features, acute-phase responses, and B-cell activity. Although effective in disease control, both TNFi and IL6i may disrupt immune homeostasis and are suspected to differentially trigger SADs through unique effects on cytokine signaling and immune regulation (12–14, 18).

Prior studies have documented rare but serious autoimmune sequelae associated with bDMARDs. TNFi-induced lupus-like syndromes, demyelinating disorders, and autoimmune cytopenias have been reported, while IL6i has been associated with Drug Reaction With Eosinophilia and Systemic Symptoms (DRESS) and unusual severe pulmonary disease, including diffuse lung disease and pulmonary hypertension (11, 14, 19–21). Despite the clinical efficacy of biologics, these immunomodulators have raised concerns about paradoxical autoimmune effects, especially the underlying mechanisms involving shifts in Th17/Treg cell balance, dysregulated antigen presentation, and B-cell hyperactivity—processes particularly sensitive in pediatric populations whose immune systems are still developing (22). Despite post-marketing surveillance by regulatory bodies such as the FDA (Food and Drug Administration) and EMA (European Medicines Agency), much of the current safety data originates from adult cohorts, limiting its applicability to children. Real-world data platforms now offer the ability to conduct large-scale retrospective analyses with real-time propensity matching, enabling more accurate and generalizable insights into pediatric outcomes (23).

To date, no large, matched-cohort studies have directly compared the risk of systemic autoimmune diseases in children with JA treated with TNFi versus IL6i using real-world evidence. Most existing literature focuses on drug efficacy or adult safety data, failing to capture nuanced risks in pediatric populations or to control adequately for confounders such as age, sex, baseline disease activity, and co-medications. This research aims to address these gaps by leveraging TriNetX to conduct a comparative safety evaluation of TNFi and IL6i, with a specific focus on the development of SADs in JIA patients.

In this study, we utilized a retrospective cohort design using the TriNetX Research Network, which compiles longitudinal, de-identified clinical data from over 120 million patient records. Pediatric patients (≤18 years) diagnosed with JIA and newly treated with either TNFi or IL6i from January 1, 2013, to December 31, 2024, were identified. One-to-one propensity score matching was applied to minimize baseline imbalances. The primary outcome was the incidence of SADs, defined using a composite of ICD-coded diagnoses. Hazard ratios (HRs) were estimated via Cox proportional hazards models. To ensure robustness, subgroup analyses were stratified by age, sex, and concomitant immunosuppressive use, and three different sensitivity models were implemented. This approach provides a comprehensive, real-world assessment of autoimmune risk associated with two commonly prescribed biologics in pediatric rheumatology.

## 2 Method

### 2.1 Data source

This retrospective cohort study utilized de-identified electronic health records from TriNetX, a U.S.-based collaborative network encompassing data on approximately 122 million patients. The dataset includes information from various healthcare environments, such as hospitals, primary care facilities, and specialty clinics, and contains anonymized patient records. Key data components include demographic information, diagnoses coded using the International Classification of Diseases, Tenth Revision, Clinical Modification (ICD-10-CM), medications classified by the Anatomical Therapeutic Chemical (ATC) system and standardized via RxNorm, procedures recorded with ICD-10-PCS, Current Procedural Terminology (CPT), and Systematized Nomenclature of Medicine (SNOMED), as well as laboratory test results coded with Logical Observation Identifier Names and Codes (LNC).

TriNetX serves as a global federated platform for real-world data integration and analysis, supporting diverse research initiatives (23). This study was exempted from informed consent requirements under Section §164.514(a) of the HIPAA Privacy Rule due to its reliance on anonymized secondary data without direct interaction with human subjects. The de-identification process adhered to Section §164.514(b)(1) of the HIPAA Privacy Rule, verified by a qualified expert. Ethical approval was granted by the Institutional Review Board of Chung Shan Medical University Hospital (IRB No.: CS2-21176).

### 2.2 Study design

**Figure 1:**
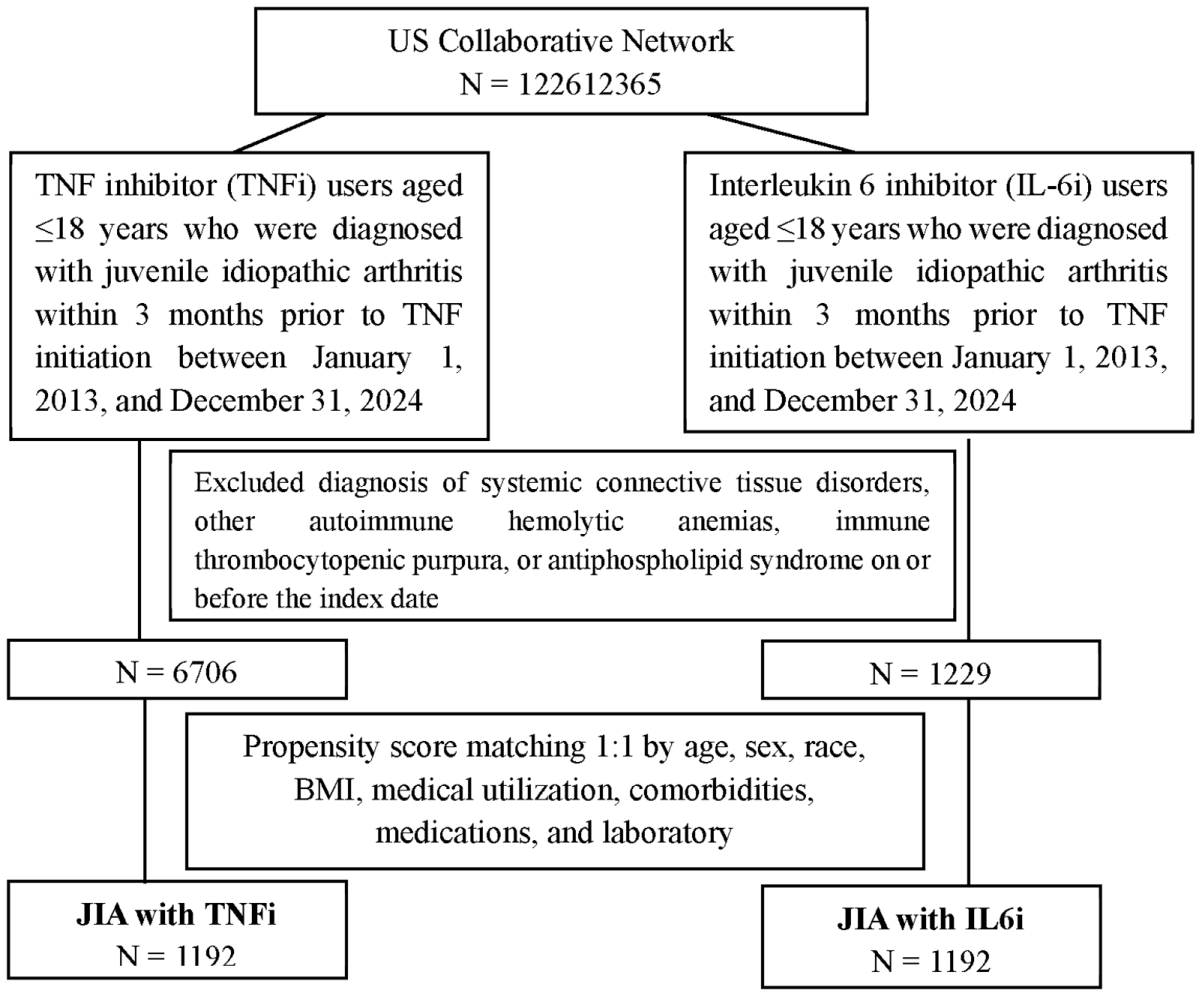
Flow chart of subject selection of JIA (Juvenile idiopathic arthritis) treated with TNFi versus IL-6i.

**Figure 1** illustrates the flowchart for cohort construction. The exposed study population consisted of patients aged ≤18 who initiated TNFi therapy between January 1, 2013, and December 31, 2024, and diagnosed with juvenile idiopathic arthritis (ICD-10-CM: M08) within 3 months prior to TNFi initiation. The TNFi of interest included etanercept (RxNorm: 214555), infliximab (RxNorm: 191831), adalimumab (RxNorm: 327361), certolizumab (RxNorm: 709271), and golimumab (RxNorm: 819300). The comparison study population consisted of patients aged ≤18 years who initiated IL6i therapy, specifically tocilizumab (RxNorm: 612865) or sarilumab (RxNorm: 1923319), during the same period and had a diagnosis of juvenile idiopathic arthritis within 3 months prior to IL-6i initiation. The index date was defined as the date of the first prescription of either a TNFi or an IL-6i. The primary analysis followed an intention-to-treat–like approach, defining exposure by the initial biologic received. Treatment switching is common in real-world pediatric rheumatology and may reflect disease refractoriness or intolerance; therefore, restricting the main cohort to non-switchers could introduce selection bias. To address switching rigorously, we conducted a separate non-switcher sensitivity analysis in which individuals who transitioned between TNFi and IL-6i were excluded. Both groups excluded patients with a diagnosis of systemic connective tissue disorders (ICD-10-CM: M30–M36), other autoimmune hemolytic anemias (ICD-10-CM: D59.1), immune thrombocytopenic purpura (ICD-10-CM: D69.3), or antiphospholipid syndrome (ICD-10-CM: D68.61) on or before the index date. Subgroup analyses were conducted to assess the association between TNFi and IL6i across subgroups defined by age, sex, race, asthma status, and concomitant use of NSAIDs, corticosteroids, and methotrexate. Additionally, the analysis was further restricted to patients diagnosed with juvenile rheumatoid arthritis (ICD-10-CM codes M08.0, M08.3, and M08.4), systemic juvenile arthritis (M08.2), and oligoarticular juvenile arthritis (M08.4) to evaluate the association between TNFi and IL6i use and the subsequent development of SADs. In this analysis, ‘juvenile rheumatoid arthritis’ (ICD-10-CM M08.0, M08.3, M08.4) corresponds to polyarticular subtypes of JIA. Sensitivity analyses were performed to evaluate the robustness of the association between treatment exposure and the risk of systemic autoimmune diseases using three propensity score–matched models. Model 1 matched patients 1:1 based on age, sex, race, and body mass index (BMI). Model 2 additionally accounted for medical utilization and baseline comorbidities. Model 3 further included adjustments for concomitant medications and laboratory parameters. To minimize potential confounding from treatment crossover, the cohort was further restricted to patients who remained on their initial biologic therapy without switching between tumor necrosis factor inhibitors and interleukin-6 inhibitors throughout the study period. All statistical procedures were conducted on the TriNetX platform, which integrates R 4.0.2 for advanced analyses.

Baseline characteristics included age, sex, race, body mass index (BMI), and utilization of outpatient, emergency, and inpatient medical services. Comorbidities comprised diabetes mellitus, hypertensive diseases, hyperlipidemia, asthma, atopic dermatitis, vasomotor and allergic rhinitis. Medication use encompassed nonsteroidal anti-inflammatory drugs (NSAIDs), corticosteroids, methotrexate, and hydroxychloroquine. Laboratory data included C-reactive protein, plasma, leukocytes, rheumatoid factor and HLA-B27 [presence] detected by flow cytometry (FC). Detailed codes for all variables are provided in **Supplementary Table 1**.

Propensity score matching (PSM) was implemented to address baseline differences and control for confounding factors. A 1:1 matching ratio was applied using the TriNetX platform, considering variables such as age, sex, race, BMI, medical utilization, comorbidities, medications, and laboratory markers. A greedy nearest-neighbor algorithm with a caliper of 0.1 pooled standard deviations was employed. The primary objective was to compare the risk of systemic autoimmune diseases between users of TNFi and IL6i. For analytic purposes, systemic autoimmune diseases (SADs) were grouped into two predefined categories based on shared clinical and immunopathological characteristics. *Category 1* included vasculitides and immune-mediated cytopenias—specifically polyarteritis nodosa and related conditions, Wegener’s granulomatosis, microscopic polyangiitis, necrotizing vasculopathy, autoimmune hemolytic anemia, immune thrombocytopenic purpura, and antiphospholipid syndrome. These disorders share predominant vascular-inflammatory and hematologic-autoimmune mechanisms. *Category 2* consisted of systemic connective-tissue diseases, including systemic lupus erythematosus, dermatomyositis/polymyositis, systemic sclerosis, Sjögren syndrome, and overlap syndromes, representing multi-organ systemic autoimmunity. This categorization reflects commonly recognized clinical groupings in pediatric rheumatology and facilitates interpretation of disease-specific risks in the comparative analyses. SADs included the following conditions, namely polyarteritis nodosa and related conditions (ICD-10-CM = M30), Wegener’s granulomatosis (ICD-10-CM = M31.3), microscopic polyangiitis (ICD-10-CM = M31.7), necrotizing vasculopathy, unspecified (ICD-10-CM = M31.9), other autoimmune hemolytic anemias (ICD-10-CM = D59.1), immune thrombocytopenic purpura (ICD-10-CM = D69.3), antiphospholipid syndrome (ICD-10-CM = D68.61), systemic lupus erythematosus (ICD-10-CM = M32), dermatopolymyositis (ICD-10-CM = M33), systemic sclerosis (ICD-10-CM = M34), Sjögren syndrome (ICD-10-CM = M35.0), and other overlap syndromes (ICD-10-CM = M35.1). Participants were followed until the first occurrence of systemic autoimmune diseases or their last recorded medical event within the US collaborative network.

### 2.3 Statistical analysis

Statistical analyses were performed using the TriNetX platform. Balance between matched cohorts was evaluated using standardized mean differences (SMD), with values below 0.1 indicating sufficient balance. An SMD <0.1 is commonly used to indicate adequate covariate balance in propensity-matched observational studies. Kaplan-Meier survival analysis was used to compare the cumulative incidence of systemic autoimmune diseases between the cohorts, with statistical significance assessed via the log-rank test. Cox proportional hazards models were utilized to estimate the relationship between cohort assignment and the risk of systemic autoimmune diseases, presenting Hazard ratios (HRs) with 95% confidence intervals.

## 3 Result

### 3.1 Baseline characteristics

After 1:1 PSM, each treatment group included 1,192 patients (**Figure 1**). As shown in **Table 1**. Standardized mean differences (SMDs) were below 0.1 for most variables, indicating adequate covariate balance after matching. Two variables—CRP (SMD = 0.103) and HLA-B27 positivity (SMD = 0.324)—showed slightly higher SMDs; these were retained in the model because they represent clinically relevant inflammatory markers, and the overall covariate balance remained acceptable. The mean age was similar (11·56 ± 4·80 vs. 11·61 ± 4·66 years, SMD = 0·012), and the proportion of female patients was nearly identical (76·2% vs. 75·2%, SMD = 0·023). Racial distribution, BMI, and healthcare utilization patterns, including ambulatory, emergency, and inpatient encounters, showed no substantial differences. Common comorbidities such as diabetes mellitus, hypertension, hyperlipidemia, asthma, and atopic dermatitis were rare and comparable between the groups. The use of concomitant medications, including NSAIDs, corticosteroids, methotrexate, and hydroxychloroquine, was also well balanced. Laboratory values such as C-reactive protein (CRP), leukocyte counts, rheumatoid factor levels, and HLA-B27 positivity did not differ meaningfully between the groups after matching.

**Table 1:** Demographic characteristics of JIA cohorts treated with TNFi versus IL6i.

	Before PSM			After PSM		SMD
	JIA with TNFi N = 6706	JIA with IL6i N = 1229	SMD	JIA with TNFi N = 1192	JIA with IL6i N = 1192	
**Age, Mean ± SD**	11·28 ± 4·67	11·59 ± 4·68	0·066	11·56 ± 4·80	11·61 ± 4·66	0·012
Sex						
Female	4700 (70·09)	920 (74·86)	**0·107**	908 (76·18)	896 (75·17)	0·023
Male	1994 (29·74)	307 (24·98)	**0·107**	281 (23·57)	294 (24·66)	0·025
Unknown Gender	12 (0·18)	10 (0·81)	0·090	10 (0·84)	10 (0·84)	<0·001
Race						
White	4849 (72·31)	817 (66·48)	**0·127**	814 (68·29)	792 (66·44)	0·039
Black or African American	412 (6·14)	84 (6·84)	0·028	78 (6·54)	80 (6·71)	0·007
Asian characteristic(s)	12 (0·18)	10 (0·81)	0·090	10 (0·84)	10 (0·84)	<0·001
Native Hawaiian or Other Pacific Islander	22 (0·33)	10 (0·81)	0·064	10 (0·84)	10 (0·84)	<0·001
American Indian or Alaska Native	57 (0·85)	10 (0·81)	0·004	10 (0·84)	10 (0·84)	<0·001
Other Race	541 (8·07)	151 (12·29)	**0·140**	135 (11·33)	144 (12·08)	0·023
Unknown Race	654 (9·75)	137 (11·15)	0·046	126 (10·57)	136 (11·41)	0·027
**BMI, Mean ± SD**	20·70 ± 5·94	21·80 ± 6·66	0·175	21·48 ± 6·61	21·78 ± 6·62	0·045
Medical utilization						
Ambulatory	5651 (84·27)	1079 (87·80)	**0·102**	1037 (87·00)	1044 (87·58)	0·018
Emergency	669 (9·98)	182 (14·81)	**0·147**	190 (15·94)	169 (14·18)	0·049
Inpatient Encounter	321 (4·79)	156 (12·69)	**0·283**	131 (10·99)	132 (11·07)	0·003
Comorbidities						
Diabetes mellitus	58 (0·87)	10 (0·81)	0·006	10 (0·84)	10 (0·84)	<0·001
Hypertensive diseases	42 (0·63)	37 (3·01)	**0·179**	26 (2·18)	21 (1·76)	0·030
Hyperlipidemia	39 (0·58)	18 (1·47)	0·088	12 (1·01)	10 (0·84)	0·018
Asthma	240 (3·58)	56 (4·56)	0·050	60 (5·03)	52 (4·36)	0·032
Atopic dermatitis	46 (0·69)	15 (1·22)	0·055	13 (1·09)	10 (0·84)	0·026
Vasomotor and allergic rhinitis	153 (2·28)	28 (2·28)	<0·001	30 (2·52)	28 (2·35)	0·011
Medications						
NSAIDs	3618 (53·95)	726 (59·07)	**0·103**	699 (58·64)	695 (58·31)	0·007
Corticosteroids	2477 (36·94)	736 (59·89)	**0·472**	696 (58·39)	700 (58·73)	0·007
Methotrexate	3404 (50·76)	537 (43·69)	**0·142**	561 (47·06)	528 (44·30)	0·056
Hydroxychloroquine	184 (2·74)	53 (4·31)	0·085	62 (5·20)	49 (4·11)	0·052
Laboratory						
C reactive protein [Mass/volume] in Serum, Plasma or Blood	20·34 ± 30·39	36·35 ± 56·18	**0·354**	28·54 ± 39·20	33·28 ± 51·82	**0·103**
<30	1742 (25·98)	443 (36·05)	**0·219**	431 (36·16)	416 (34·90)	0·026
≥30	569 (8·49)	279 (22·70)	**0·400**	248 (20·81)	249 (20·89)	0·002
Leukocytes [#/volume] in Blood	8·96 ± 69·13	14·43 ± 160·30	0·044	8·14 ± 2·78	14·47 ± 163·72	0·055
Rheumatoid factor [Units/volume] in Serum or Plasma	26·61 ± 24·42	24·76 ± 23·85	0·077	23·87 ± 19·92	24·76 ± 23·85	0·041
<20	140 (2·09)	14 (1·14)	0·075	10 (0·84)	14 (1·17)	0·034
≥20	206 (3·07)	20 (1·63)	0·095	21 (1·76)	20 (1·68)	0·006
HLA-B27 [Presence] by Flow cytometry (FC)	33,112·77 ± 575,435·25	526,315·60 ± 2,294,157·20	**0·295**	0·33 ± 0·62	526,315·60 ± 2,294,157·20	**0·324**
>0	300 (4·47)	16 (1·30)	**0·190**	14 (1·17)	16 (1·34)	0·015

*JIA, Juvenile idiopathic arthritis; TNFi, Tumor necrosis factor inhibitors; IL-6i, interleukin-6 inhibitors.

*Bold font indicates that the standardized difference was greater than 0·1.

*If the number of patients is less than or equal to 10, the results are shown as a count of 10.

*SD, Standard deviation; SMD, Standardized mean difference.

*NSAIDs, Nonsteroidal Anti-Inflammatory Drugs.

*Propensity score matching (PSM) was performed on age at the index date, sex, race, BMI, medical utilization, comorbidities, medications, and laboratory markers.

### 3.2 Risk of systemic autoimmune diseases

**Figure 2:**
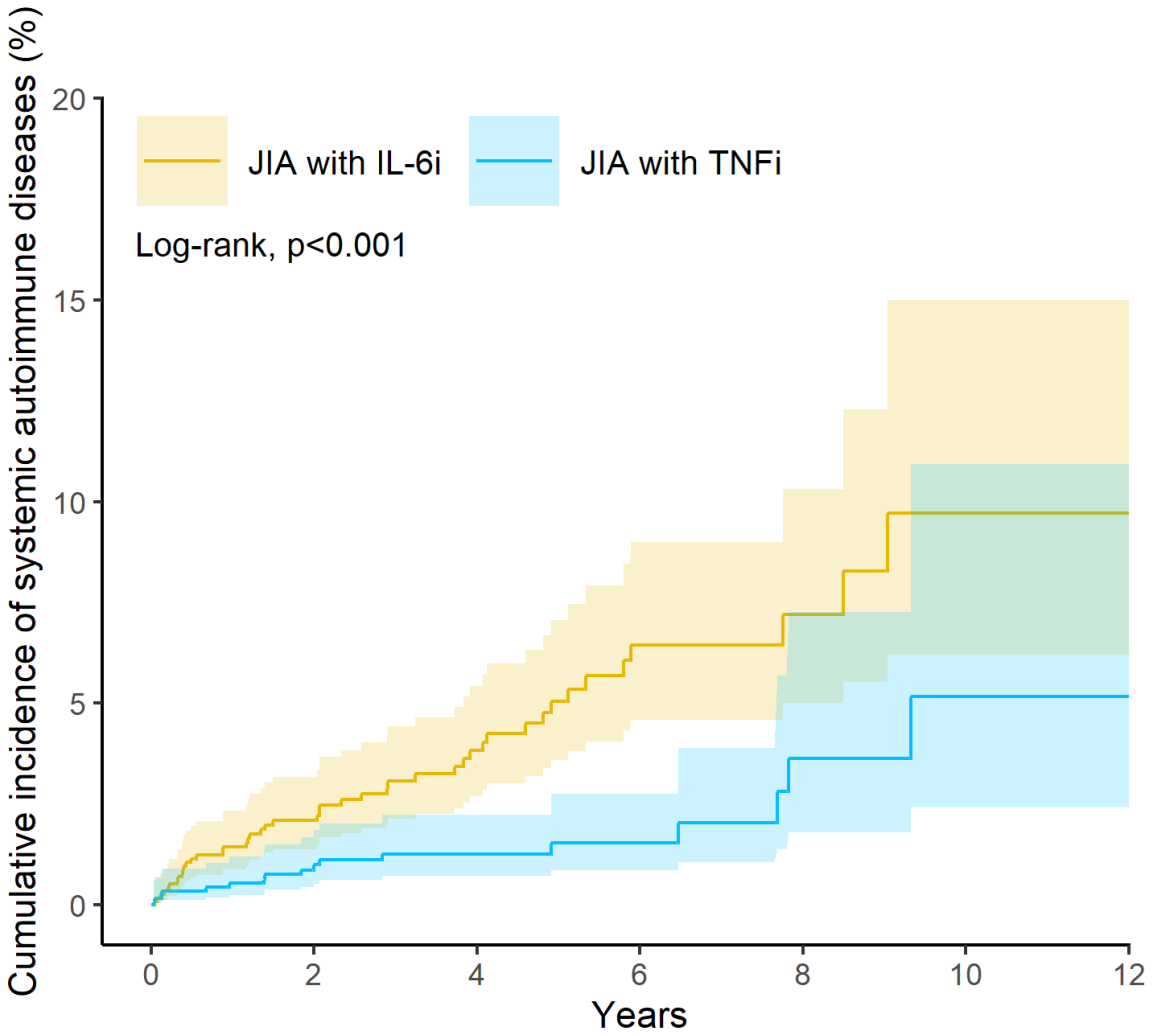
Kaplan-Meier plot for risk of systemic autoimmune diseases.

The incidence of systemic autoimmune diseases was significantly lower in the TNFi group than in the IL6i group, with 17 and 45 events observed, respectively. As presented in **Table 2**, this corresponded to a hazard ratio (HR) of 0·37 (95% confidence interval [CI], 0·20-0·63). Disease-specific analyses indicated that polyarteritis nodosa and systemic sclerosis occurred exclusively in IL6i-treated patients (n = 10 each), precluding HR estimation. For other conditions within the same categories, including systemic lupus erythematosus, dermatopolymyositis, Sjögren syndrome, and overlap syndromes, the TNFi group consistently demonstrated a lower or similar risk. The HR for Category 1 conditions was 0·48 (95% CI, 0·14-1·60), and for Category 2 conditions was 0·35 (95% CI, 0·18-0·64). The cumulative incidence curves illustrated in **Figure 2** demonstrate the reduced risk of systemic autoimmune diseases among TNFi-treated patients.

**Table 2:** Risk of systemic autoimmune diseases exposed to JIA treated with TNFi compared to JIA with IL6i.

	No· of event		HR (95% CI)
	JIA with TNFi N = 1192	JIA with IL6i N = 1192	
**Systemic autoimmune diseases**	17	45	**0·37 (0·20-0·63)**
**Category 1**	10	10	0·48 (0·14-1·60)
Polyarteritis nodosa and related conditions	0	10	N/A
Wegener’s granulomatosis/microscopic polyangiitis	0	0	N/A
Necrotizing vasculopathy, unspecified	0	0	N/A
Other autoimmune hemolytic anemias	10	10	0·98 (0·06-15·60)
Immune thrombocytopenic purpura	10	10	0·99 (0·14-7·05)
Antiphospholipid syndrome	10	10	0·46 (0·04-5·06)
**Category 2**	14	39	**0·35 (0·18-0·64)**
Systemic lupus erythematosus	10	12	0·65 (0·26-1·59)
Dermatopolymyositis	10	10	0·37 (0·09-1·38)
Systemic sclerosis	0	10	N/A
Sjögren syndrome	10	10	0·28 (0·05-1·32)
Other overlap syndromes	10	10	0·20 (0·04-0·90)

*HR, Hazard ratio. 95% CI, 95% confidence interval.

*If the patient’s count is 1-10, the results indicate a count of 10.

*N/A, Not Applicable.

*Bold font indicates statistical significance.

TriNetX privacy policy requires masking of event counts between 1 and 10, which are uniformly displayed as ‘10’ in accordance with platform rules. Statistical analyses were performed using the underlying unmasked values. Category 1 includes vasculitides and immune-mediated cytopenias; Category 2 includes systemic connective-tissue autoimmune diseases.

### 3.3 Subgroup analysis of the risk of systemic autoimmune diseases

**Figure 3:**
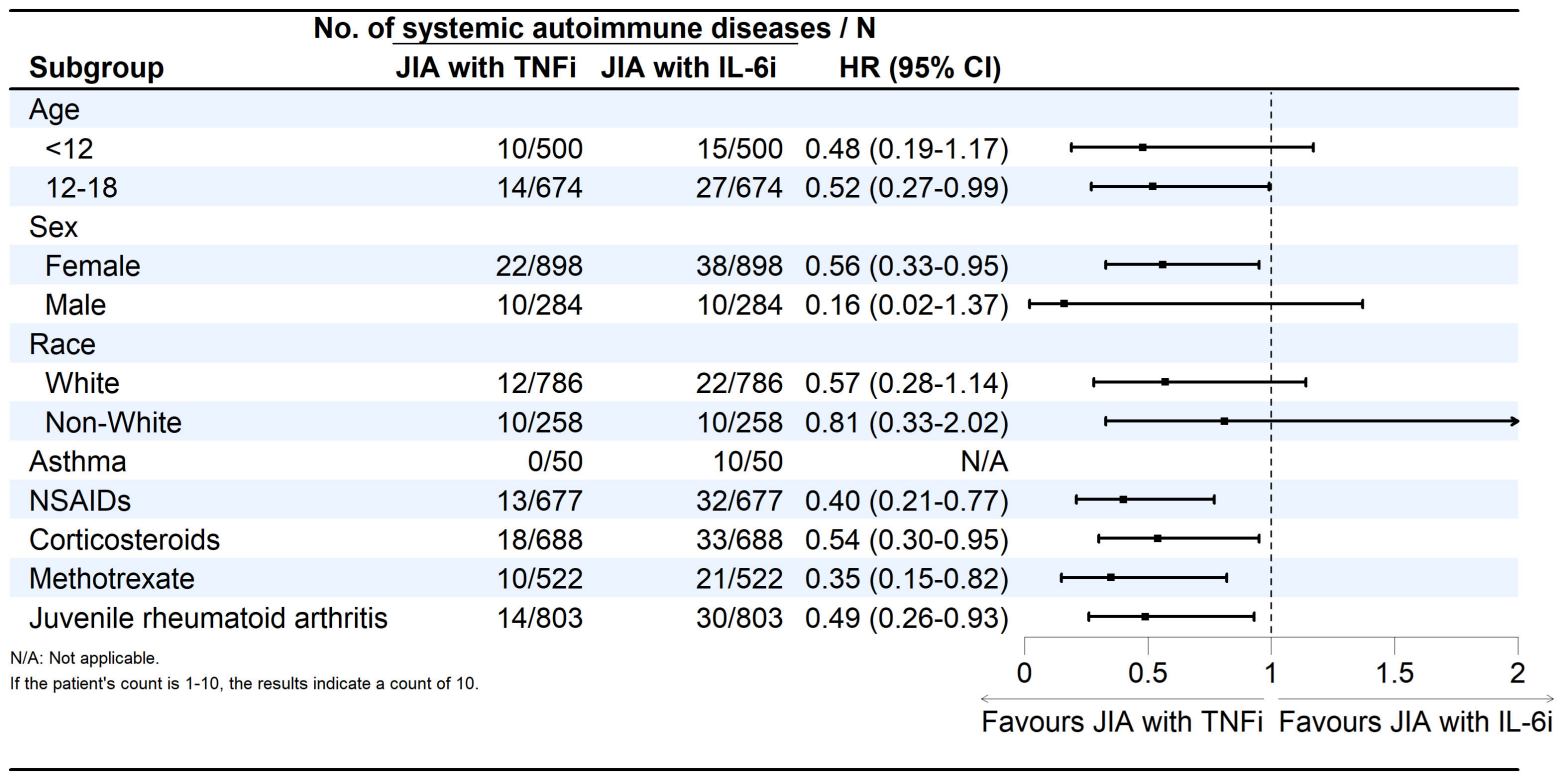
Forest plot of risk of systemic autoimmune diseases exposed to JIA treated with TNFi compared to JIA with IL6i.

As shown in **Supplementary Table 2** (**Figure 3**), subgroup analyses revealed consistent protective effects of TNFi across various strata. Among patients aged 12 to 18, the HR was 0·52 (95% CI, 0·27-0·99), while among those under 12 years, the HR was 0·48 (95% CI, 0·19-1·17). In female patients, the HR was 0·56 (95% CI, 0·33-0·95), whereas in male patients, no significant association was observed (HR, 0·16; 95% CI, 0·02-1·37). Among patients using NSAIDs, corticosteroids, or methotrexate, TNFi was associated with reduced risks: HRs were 0·40 (95% CI, 0·21-0·77), 0·54 (95% CI, 0·30-0·95), and 0·35 (95% CI, 0·15-0·82), respectively. In patients diagnosed with juvenile rheumatoid arthritis, TNFi was associated with a lower risk compared to IL6i (HR, 0·49; 95% CI, 0·26-0·93). However, there were no differences between patients with systemic juvenile arthritis and those with oligoarticular juvenile arthritis.

### 3.4 Sensitivity analyses evaluating the risk of systemic autoimmune diseases

**Table 3** summarizes the results of multiple sensitivity analyses, confirming the robustness of the observed associations. Model 1, adjusting for age, sex, race, and BMI, showed an HR of 0·43 (95% CI, 0·25-0·72). Model 2, which additionally accounted for medical utilization and comorbidities, yielded an HR of 0·46 (95% CI, 0·27-0·77). Model 3, which incorporated medication use and laboratory data, produced an HR of 0·37 (95% CI, 0·21-0·64). In the non-switcher sensitivity cohort (898 per group), the reduced risk associated with TNFi remained evident and even stronger (HR 0.28; 95% CI 0.13–0.62), supporting that treatment switching did not account for the main findings. The risk reduction was more pronounced among non-switchers, who remained on their initial biologic throughout follow-up (HR, 0·28; 95% CI, 0·13-0·62), reinforcing the consistent association between TNFi exposure and a lower risk of systemic autoimmune diseases.

**Table 3:** Sensitivity analyses evaluating the risk of systemic autoimmune diseases between JIA treated with TNFi and JIA with IL-6i using alternative models.

	JIA with TNFi		JIA with IL6i		
	N	No· of event	N	No·of event	HR (95% CI)
Model 1	1206	20	1206	46	**0·43 (0·25-0·72)**
Model 2	1198	21	1198	46	**0·46 (0·27-0·77)**
Model 3	1192	17	1192	45	**0·37 (0·20-0·63)**
Non-switchers	898	10	898	28	**0·28 (0·13-0·62)**

*Model 1: Propensity score matching 1:1 by age, sex, race, and BMI.

*Model 2: Propensity score matching 1:1 by age, sex, race, BMI, medical utilization, and comorbidities.

*Model 3: Propensity score matching 1:1 by age, sex, race, BMI, medical utilization, comorbidities, medications, and laboratory markers.

*If the patient’s count is 1-10, the results indicate a count of 10.

*Bold values indicate significant protection for JIA with TNFi.

## 4 Discussion

In this large-scale, retrospective cohort study utilizing propensity score-matched data from 2,384 JIA patients, we observed a significantly lower risk of SADs among those treated with TNFi compared to those receiving IL6i. Specifically, the incidence of SADs was 63% lower in the TNFi group, with a hazard ratio (HR) of 0·37 (95% CI 0·20–0·63). Although treatment switching is a potential source of exposure misclassification, the consistency of results across the intention-to-treat–like primary analysis, three PSM models, and the strict non-switcher cohort indicates that the observed difference in SAD risk is robust and unlikely to be driven by switching behavior. Notably, certain autoimmune diseases, such as polyarteritis nodosa and systemic sclerosis, were observed exclusively in the IL6i-treated cohort, whereas these conditions were absent in the TNFi group. The protective effect of TNFi was consistent across various clinically relevant subgroups, including different age categories, sexes, and concomitant medication use (such as NSAIDs, corticosteroids, and methotrexate). In subgroup analyses stratified by concomitant therapy, the reduced risk associated with TNFi persisted. Because NSAIDs, corticosteroids, and MTX are used similarly across both biologic classes and are not determinants of treatment assignment, these findings likely reflect intrinsic differences in immunopathologic mechanisms between TNF-α inhibition and IL-6 blockade rather than medication interactions. Sensitivity analyses, including alternative propensity score models and restriction to non-switcher populations, confirmed the robustness of these findings, with hazard ratios ranging from 0·28 to 0·46. Furthermore, the Kaplan-Meier survival curves demonstrated a clear divergence in the cumulative incidence of systemic autoimmune diseases over time, favoring TNFi therapy. These results collectively highlight a distinct and clinically meaningful difference in the autoimmune safety profiles of TNFi and IL6i in pediatric JIA patients, suggesting that TNFi may offer a safer long-term option for patients at risk of developing systemic autoimmune complications.

Our findings of a 63% reduced risk of systemic autoimmune diseases with TNFi versus IL6i align with and extend prior observations on cytokine modulation and autoimmune pathways in JIA. The exclusive occurrence of polyarteritis nodosa and systemic sclerosis in IL6i-treated patients contrasts with historical case reports of IL-6 blockade efficacy in refractory polyarteritis nodosa (PAN) (24), highlighting the dual role of IL-6 inhibition in autoimmune regulation. Meanwhile, the consistent protective effect of TNFi across subgroups corroborates mechanistic insights from cytokine profiling studies (25), while challenging conventional safety paradigms derived from efficacy-focused systematic reviews (26). Below, we contextualize each key result within the broader literature and defend our findings against potential contradictions.

### 4.1 TNFi protection against systemic autoimmune diseases

Our observation of reduced autoimmune risk with TNFi (HR 0·37) aligns with pharmacovigilance data showing TNFi-associated lupus risks are agent-specific and manageable (27). While Moulis et al. (27) reported higher lupus risk with monoclonal TNFi (infliximab, adalimumab) versus etanercept, our study aggregates all TNFi, potentially diluting agent-specific risks. However, the overall protective trend against broad autoimmune outcomes (beyond lupus) suggests TNFi may suppress systemic autoimmunity through pathways such as IL-6/IL-8 downregulation (25). Walters et al. (25) demonstrated that TNFi therapy reduces IL-6 and IL-8 levels in JIA patients, which may explain the lower incidence of IL-6-driven vasculopathies (e.g., PAN) in our TNFi cohort. This contrasts with IL6i’s inability to modulate Th17/Treg balance (28), a pathway critical in systemic autoimmunity.

Despite systematic reviews questioning TNFi efficacy in systemic JIA (26), our focus on autoimmune safety rather than efficacy reveals a distinct advantage for TNFi. The discordance arises because efficacy studies prioritize joint-specific outcomes, whereas autoimmune complications manifest via divergent pathways. Our real-world data complement RCTs by capturing long-term autoimmune risks underrepresented in controlled trials.

### 4.2 IL6i-associated polyarteritis nodosa and systemic sclerosis

The exclusive occurrence of PAN and systemic sclerosis in IL6i-treated patients appears paradoxical given case reports of tocilizumab efficacy in refractory PAN (24). However, Saunier et al. (24) described IL-6 blockade in *adults* with *pre-existing* PAN, whereas our cohort involved *de novo* autoimmune events in *pediatric* JIA patients. This suggests IL6i may suppress established PAN via acute-phase protein normalization (29) while paradoxically inducing vasculopathy in predisposed individuals through Th17 dysregulation (28). IL-6 is critical for Treg/Th17 balance, and its inhibition may destabilize immune tolerance, particularly in children with developing immune systems. Similarly, the link between IL6i and systemic sclerosis aligns with IL-6’s role in fibroblast activation and fibrosis (28), pathways less prominent in TNFi’s mechanism.

While IL6i’s efficacy in adult PAN (24) seems contradictory, the pediatric JIA population’s unique immunobiology, marked by heightened IL-6 signaling in systemic subtypes, may predispose to IL6i-induced vasculopathy. Our findings emphasize that IL-6’s role differs between disease contexts: therapeutic in chronic, refractory autoimmunity but potentially harmful when applied to developing or subclinical autoimmunity.

### 4.3 Subgroup consistency and mechanistic insights

The persistent TNFi protection across age, sex, and concomitant medications mirrors sex-specific cytokine responses observed in JIA. Females showed stronger TNFi protection (HR 0·56 vs. male HR 0·16), consistent withWalters et al. (25), where TNFi more effectively suppressed IL-6 in females. The protective effect in methotrexate users (HR 0·35) may reflect synergism between TNFi and methotrexate in suppressing IL-17, a cytokine elevated in etanercept-treated patients (25). Notably, IL-17 promotes neutrophil recruitment and vascular inflammation, potentially exacerbating IL6i-associated vasculopathies.

Critics may argue that residual confounding persists despite propensity score matching. However, our sensitivity analyses (HR 0·28–0·46) and consistency across subgroups mitigate this concern. The mechanistic plausibility, TNFi’s suppression of IL-6/IL-8 (25) versus IL6i’s Th17/Treg disruption (28), strengthens the biological validity of our findings. But further studies are warranted for further exploration of this mechanistic insights.

### 4.4 Contrasts with prior safety literature

Our results challenge the perception of IL6i as uniformly safe in JIA. While Wobma et al. (21) linked IL-6 inhibition to eosinophilia and lung disease, we extend these observations to broader autoimmune outcomes. Conversely, Yoshizaki et al. (29) emphasized IL6i’s superiority in normalizing acute-phase proteins (e.g., CRP), but our data suggest this benefit may come at the cost of autoimmune activation. Similarly, systematic reviews of TNFi (26) prioritized infection and malignancy risks over autoimmune outcomes, creating an evidence gap that our study fills.

The TriNetX database’s scale (122 million patients) and longitudinal design provide statistical power to detect rare autoimmune events missed in smaller cohorts. While RCTs (26) focus on short-term efficacy, our real-world data capture the latent autoimmune risks critical for chronic JIA management.

### 4.5 Reconciling apparent contradictions

The dual role of IL-6 blockade—therapeutic in refractory PAN (24) but pathogenic in *de novo* JIA—highlights the context-dependent nature of cytokine inhibition. IL-6’s pleiotropic effects mean its blockade may suppress inflammation in established autoimmunity while destabilizing immune homeostasis in predisposed individuals. This duality parallels TNFi’s role in triggering lupus (27) despite overall autoimmune protection, underscoring the complexity of cytokine networks.

Our study does not negate IL6i’s value in JIA but advocates for risk stratification. Patients with familial autoimmunity or baseline eosinophilia (21) may benefit from TNFi, while IL6i remains preferred for TNFi-resistant cases.

Our results are consistent with mechanistic studies of cytokine modulation (25, 28) yet reveal underappreciated autoimmune risks of IL6i in pediatric populations. By contextualizing these findings within the literature, we advance a paradigm shift: TNFi’s systemic immune modulation offers broader autoimmune protection, while IL6i’s targeted effects carry context-specific risks. This justifies personalized biologic selection based on autoimmune predisposition, a strategy absent in current guidelines.

This study contribute**s** meaningfully to pediatric rheumatology and pharmacovigilance literature in several ways. First, it is among the largest comparative analyses to date evaluating biologic-related SAD risk in children and adolescents with juvenile arthritis. Second, it leverages the expansive and standardized TriNetX database, enhancing data quality and external validity. Third, using three-tiered propensity score matching, subgroup analyses, and sensitivity testing addresses residual confounding more rigorously than previous research. Finally, the study provides disease-specific insights into autoimmune risks, revealing that conditions like systemic sclerosis and polyarteritis nodosa may be more common in IL6i-treated patients. These findings may influence clinical decision-making by helping physicians weigh the immunologic safety of TNFi versus IL6i, particularly in long-term therapeutic planning for pediatric patients.

Several limitations must be acknowledged. First, the observational design precludes definitive causal inferences despite rigorous matching. Residual confounding by unmeasured factors, such as family history of autoimmune disease or adherence patterns, cannot be excluded. Second, administrative coding may under- or overestimate the true incidence of SADs, although TriNetX employs harmonized data curation. Third, this analysis focuses on U.S.-based data, potentially limiting generalizability to other healthcare systems. Additionally, the IL6i cohort was smaller at baseline, raising the possibility of reduced statistical power for rarer outcomes. Future research should include prospective registries or randomized controlled trials (RCTs) to validate these findings. Molecular and immunophenotyping studies could further elucidate the mechanistic pathways linking biologic class to autoimmune risk. Finally, long-term post-marketing surveillance and pharmacogenomic integration could optimize biologic selection for pediatric autoimmune disease management.

## 5 Conclusion

This study demonstrates that juvenile idiopathic arthritis patients treated with TNFi have a significantly lower risk of developing systemic autoimmune diseases compared to those receiving IL6i. Notably, vasculopathic conditions such as polyarteritis nodosa and systemic sclerosis were observed exclusively in the IL6i group. The protective effect of TNFi was consistent across age, sex, and concomitant medication subgroups, with robust findings confirmed by sensitivity analyses. These results highlight important differences in the autoimmune safety profiles of TNFi and IL6i, emphasizing the need for personalized biologic selection and vigilant monitoring, especially for IL6i-treated patients. This study fills a critical knowledge gap and provides real-world evidence to guide safer, more effective biologic therapy choices in pediatric rheumatology, ultimately aiming to improve long-term outcomes for children with JIA.

## Data Availability

The data analyzed in this study is subject to the following licenses/restrictions: The data used in this study were obtained from the TriNetX Research Network, a global, federated health research platform that aggregates de-identified electronic medical records from participating healthcare organizations. Access to the TriNetX dataset is subject to strict contractual and institutional restrictions. Researchers cannot download, copy, or share individual-level data outside the TriNetX environment. All analyses must be performed within the secure TriNetX platform, and only aggregate, de-identified results may be exported in accordance with TriNetX and participating institutions’ data governance policies. Therefore, the dataset itself cannot be made publicly available. Requests to access these datasets should be directed to J-CW, jccwei@gmail.com.

## Supplementary material

Supplementary Table 1 Code for covariates of JIA cohorts.

Supplementary Table 2 Subgroup analysis of risk of systemic autoimmune diseases exposed to JIA treated with TNFi compared to JIA with IL6i.
