# Supplementary material for: Comparative risk of systemic autoimmune diseases in juvenile idiopathic arthritis treated with TNF-α or IL-6 inhibitors: a real-world cohort study
Source: Front Immunol. 2026 Jan 13;16:1744226. doi: 10.3389/fimmu.2025.1744226 (PMC12835221; doi:10.3389/fimmu.2025.1744226)
Supplement: Supplementary Table 1 — Code for covariates of JIA cohorts. [file Table1.docx]

**Supplementary Materials**

**Table of Content**

Supplementary Table 1. Code for covariates of JIA cohorts.

Supplementary Table 2. Subgroup analysis of risk of systemic autoimmune diseases exposed to JIA treated with TNFi compared to JIA with IL6i.

**Supplementary Table 1.** Code for covariates of JIA cohorts.

| Item | Code |
| --- | --- |
| **Comorbidities** |  |
| Diabetes mellitus | ICD-10-CM: E08-E13 |
| Hypertensive diseases | ICD-10-CM: I10-I1A |
| Hyperlipidemia | ICD-10-CM: E78 |
| Asthma | ICD-10-CM: J45 |
| Atopic dermatitis | ICD-10-CM: L20 |
| Vasomotor and allergic rhinitis | ICD-10-CM: J30 |
| **Medications** |  |
| Nonsteroidal anti-inflammatory drugs (NSAIDs) | ATC: M01A |
| Corticosteroids | ATC: H02 |
| Methotrexate | RxNorm: 6851 |
| Hydroxychloroquine | RxNorm: 5521 |
| **Laboratory** |  |
| C reactive protein [Mass/volume] in Serum, Plasma or Blood | TNX: 9063 |
| Leukocytes [#/volume] in Blood | TNX: 9015 |
| Rheumatoid factor [Units/volume] in Serum or Plasma | TNX: 9070 |
| HLA-B27 [Presence] by Flow cytometry (FC) | LOINC: 26028-1 |

*ICD-10-CM: International Classification of Diseases, 10th Revision, Clinical Modification,

*ATC: Anatomical Therapeutic Chemical Classification,

*LOINC: Logical Observation Identifiers Names and Codes,

*RxNorm: Normalized Naming System for Drugs,

*TNX: TrinetX curated.

**Supplementary Table 2.** Subgroup analysis of risk of systemic autoimmune diseases exposed to JIA treated with TNFi compared to JIA with IL6i.

|  | JIA with TNFi | | JIA with IL6i | |  |
| --- | --- | --- | --- | --- | --- |
|  | N | No. of event | N | No. of event | HR (95% CI) |
| Age |  |  |  |  |  |
| <12 | 500 | 10 | 500 | 15 | 0.48 (0.19-1.17) |
| 12-18 | 674 | 14 | 674 | 27 | **0.52 (0.27-0.99)** |
| Sex |  |  |  |  |  |
| Female | 898 | 22 | 898 | 38 | **0.56 (0.33-0.95)** |
| Male | 284 | 10 | 284 | 10 | 0.16 (0.02-1.37) |
| Race |  |  |  |  |  |
| White | 786 | 12 | 786 | 22 | 0.57 (0.28-1.14) |
| Non-White | 258 | 10 | 258 | 10 | 0.81 (0.33-2.02) |
| Asthma | 50 | 0 | 50 | 10 | N/A |
| NSAIDs | 677 | 13 | 677 | 32 | **0.40 (0.21-0.77)** |
| Corticosteroids | 688 | 18 | 688 | 33 | **0.54 (0.30-0.95)** |
| Methotrexate | 522 | 10 | 522 | 21 | **0.35 (0.15-0.82)** |
| Juvenile rheumatoid arthritis | 803 | 14 | 803 | 30 | **0.49 (0.26-0.93)** |
| Systemic Juvenile arthritis | 64 | 10 | 64 | 10 | 1.32 (0.30-5.90) |
| Oligoarticular Juvenile arthritis | 204 | 10 | 204 | 10 | 0.88 (0.18-4.38) |

*HR: Hazard ratio. 95% CI: 95% confidence interval

*If the patient's count is 1-10, the results indicate a count of 10.

*N/A: Not Applicable.

*Bold font indicates statistical significance.
